# Long-Term Changes in Retinal Nerve Fiber Layer Thickness after Vitrectomy for Epiretinal Membrane Using Optical Coherence Tomography Images

**DOI:** 10.3390/life13091804

**Published:** 2023-08-24

**Authors:** Ki Woong Bae, Dong Ik Kim, Daniel Duck-Jin Hwang

**Affiliations:** 1 Department of Ophthalmology, Hangil Eye Hospital, Incheon 21388, Republic of Korea; kiwoonge_e@naver.com (K.W.B.); dongikey@naver.com (D.I.K.); 2 Department of Ophthalmology, Nowon Eulji Medical Center, Seoul 01830, Republic of Korea; 3 Department of Ophthalmology, Eulji University College of Medicine, Seoul 01830, Republic of Korea; 4 Department of Ophthalmology, Catholic Kwandong University College of Medicine, Incheon 22711, Republic of Korea

**Keywords:** epiretinal membrane, optical coherence tomography, retinal nerve fiber layer, vitrectomy

## Abstract

This study investigated the long-term effects of epiretinal membrane (ERM) surgery on peripapillary retinal nerve fiber layer (RNFL) thickness using optical coherence tomography (OCT) images. We included 30 patients with idiopathic ERM who underwent a vitrectomy for ERM removal with internal limiting membrane peeling. The patients were followed up for 5 years after surgery, and their medical records were reviewed for best-corrected visual acuity (BCVA) and OCT parameters. The study population comprised 24 females (80.0%), and the mean age was 65.4 ± 7.2 years. The baseline BCVA significantly improved from 0.28 ± 0.24 to 0.12 ± 0.09 logMAR (*p* < 0.001) 1 year after surgery and continued to improve for 5 years after surgery. The peripapillary RNFL thickness initially increased after surgery and then gradually decreased. The peripapillary RNFL thicknesses of the global and temporal sectors showed significant reductions 2 years after surgery, whereas those of the nasal sectors did not significantly change. The peripapillary RNFL thickness was thinner in the global and temporal areas of the operated eyes than in those of the fellow eyes 4 and 5 years after surgery. In conclusion, peripapillary RNFL thicknesses decreased in the global and temporal areas after ERM surgery, whereas peripapillary RNFL thicknesses in the nasal sectors did not change significantly during the long-term follow-up.

## 1. Introduction

Epiretinal membrane (ERM) is a prevalent macular disorder primarily found in the older population [1], often resulting in conditions such as metamorphopsia or decreased vision [2,3]. The standard treatment for symptomatic patients has evolved into pars plana vitrectomy (PPV) and ERM removal, procedures that have shown efficacy in numerous studies [4,5]. To prevent ERM recurrence and ensure complete ERM removal, surgeons often undertake an internal limiting membrane (ILM) peeling during vitrectomy [6,7,8]. Several studies have reported the surgical outcomes of ERM surgery. Some authors reported long-term postoperative data on visual function and foveal structure beyond 5 years [9,10], indicating that the long-term prognosis of ERM surgery is fair.

Among the key considerations in the evaluation of ERM surgery is the ganglion cell complex (GCC), which consists of the three innermost retinal layers, including the retinal nerve fiber layer (RNFL), ganglion cell layer (GCL), and inner plexiform layer (IPL) [11]. The RNFL, primarily composed of the axons of retinal ganglion cells (RGCs), often undergoes thickness alterations as a result of ERM surgery [12]. These RGCs provide critical insights into retinal damage, being the first cells to react to ischemic changes and demonstrating a notable concentration on the macula [13,14]. Another essential factor is the ILM, which lies adjacent to the GCC, suggesting that ILM peeling during vitrectomy may significantly alter the architecture of the inner retinal layer [15,16].

Recent studies have shown that temporal changes in peripapillary RNFL thickness occur in patients with ERM after vitrectomy and ILM peeling; however, the number of reports is limited, and long-term follow-up results are scarce [17,18,19,20,21]. Therefore, we investigated the long-term changes in the peripapillary RNFL thickness in patients with ERM after surgery.

## 2. Materials and Methods

This retrospective study was conducted at Hangil Eye Hospital Retinal Clinic. The study was conducted following the principles of the Declaration of Helsinki. The Institutional Review Board (IRB) of Hangil Eye Hospital approved this study (IRB No. 22002) and waived the requirement for informed consent from the study participants due to the retrospective nature of the study. Patient records from January 2014 to March 2022 were reviewed for idiopathic ERM treated with PPV and ERM removal.

### 2.1. Study Participants

Patients with idiopathic ERM resulting in visual disturbances, including vision deterioration and/or metamorphopsia, were enrolled in this study. The exclusion criteria were as follows: (1) concomitant retinal diseases, including diabetic retinopathy, diabetic macular edema, age-related macular degeneration, retinal vascular occlusion, and uveitis; (2) ocular conditions that affect RNFL, such as glaucoma, optic neuropathy, and history of laser photocoagulation; (3) history of intraocular surgery, excluding cataract extraction; (4) cases of inappropriate spectral domain optical coherence tomography (SD-OCT) images (due to severe media opacity, incomplete ocular examinations, or segmentation errors); (5) extensive refractive errors exceeding ±4 diopters; and (6) cases of intraoperative gas tamponade, a procedure that can affect postoperative RNFL. In cases with bilateral diseases, only the eye that had less severe ERM was included to minimize an ERM-induced artifact during OCT measurement. Contralateral eyes that had no retinal pathology, including ERM, age-related macular degeneration, or macular hole, and did not have the intraocular conditions described in the exclusion criteria were regarded as the control group. Longitudinal changes in visual outcomes and anatomical changes in the eyes were analyzed. Additionally, the study eyes and their fellow eyes were compared.

### 2.2. Surgical Procedure

A skilled retinal surgeon (D.D.H.) performed all surgeries. Under local anesthesia, a 3-port 25-G PPV was performed using the Constellation Vision Surgical System (Alcon Surgical, Fort Worth, TX, USA). In phakic eyes, cataracts were extracted before vitrectomy. The intraocular pressure (IOP) was set at 25 mmHg and maintained during the operation. After core and peripheral vitrectomy, the ERM was removed, and the ILM was peeled off using retinal forceps. The ILM was removed for 2- or 3-disc diameters around the fovea. In cases without posterior vitreous detachment (PVD), PVD was created using ocutome and proceeded with vitrectomy. In all cases, 0.05% indocyanine green (Pulsion Medical Systems AG, Munich, Germany) was used to stain the ILM.

### 2.3. Ophthalmic Examinations

Preoperatively, patients underwent thorough ophthalmic examinations, including measurement of best-corrected visual acuity (BCVA), IOP, slit-lamp examination, and dilated fundus examination. Complete ophthalmic examinations and SD-OCT evaluations were performed at baseline and 1, 3, and 6 months and 1, 2, 3, 4, and 5 years after surgery for the study eyes and fellow eyes.

### 2.4. OCT Analysis

All participants underwent OCT evaluation using SD-OCT (Spectralis; Heidelberg Engineering, Heidelberg, Germany). The images were acquired by an experienced technician who was masked from the study subjects. A macular thickness map was obtained by the 25 horizontal raster B scans (1024 A scans per line), covering a 30° × 30° area centered on the fovea. The central macular thickness (CMT) was taken in the 1 mm diameter circle of the Early Treatment Diabetic Retinopathy Study (ETDRS) thickness map from the built-in Spectralis software, Heidelberg Eye Explorer (version 6.0.9.0). The average macular thickness (aMT) and the mean GCC thickness (mGCCT) were evaluated. The average macular thickness (aMT) was calculated using the following methods. The total macular volume (mm^3^) that is automatically presented in Spectralis software was divided by the area of the ETDRS circle (9π mm^2^) and then multiplied by 10^3^. The thickness of GCC was defined as a summation of the thicknesses of macular RNFL, GCL, and IPL. The mGCCT was calculated using the same formula. The peripapillary RNFL thickness was also automatically obtained using the equipped OCT software, Heidelberg Eye Explorer (version 6.0.9.0). The peripapillary RNFL thickness was segmented into six sectors: temporal (T, 315–45°), temporal-superior (TS, 45–90°), nasal-superior (NS, 90–135°), nasal (N, 135–225°), nasal-inferior (NI, 225–270°), and temporal-inferior (TI, 270–315°). The global peripapillary RNFL thickness was measured by averaging the 360° peripapillary RNFL thickness. OCT scans were thoroughly reviewed to ensure the accurate segmentation of retinal layers and manually corrected for cases with improper indication. For mGCCT evaluation, five eyes were excluded because they had severe segmentation errors that could not be properly corrected during the observation. The correlation was assessed for visual acuity, macular thickness, and peripapillary RNFL thickness.

### 2.5. Statistical Analysis

The demographic and clinical characteristics of the study cohort were analyzed using descriptive statistics and a commercially available software package (SPSS Statistics 25.0; IBM, Armonk, NY, USA). The mean and standard deviation are presented for continuous variables, and frequencies and percentages are used for categorical variables. After the normality of the data was evaluated, parametric tests were used for normally distributed data, and non-parametric tests were used for data that did not have a normal distribution. The significance of the difference between the variables was evaluated using a paired *t*-test or the Wilcoxon signed-rank test. Statistical significance was denoted by *p* < 0.05.

## 3. Results

**Figure 1:**
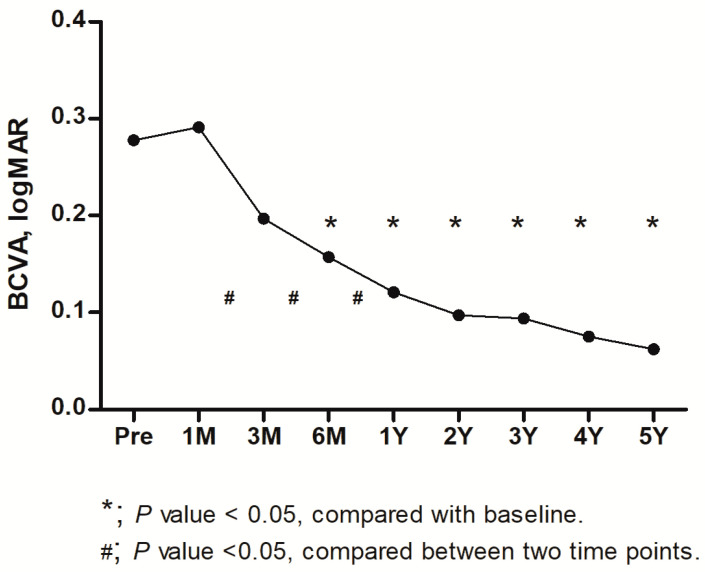
Temporal changes in the mean best corrected visual acuity (BCVA) after surgery (n = 30). The patients with epiretinal membrane showed gradual visual improvement after vitrectomy and membrane peeling during the follow-up. Compared to before surgery, the vision significantly improved 6 months after surgery (*p* = 0.006), and this visual gain continued until 5 years postoperatively. At the baseline, the mean logMAR BCVA was 0.28 ± 0.24 (range, 0.05–1.00), and it significantly improved to 0.16 ± 0.11 (range, 0.00–0.40, *p* = 0.006), 0.12 ± 0.09 (range, 0.00–0.30, *p* < 0.001), and 0.06 ± 0.08 (range, 0.00–0.30, *p* < 0.001) during the postoperative 6 months, 1 year, and 5 years, respectively. There were significant differences in vision between 1 and 3 months, 3 and 6 months, and 6 and 12 months after surgery (all *p*s < 0.05), and the visual improvement reached a plateau 1 year after the surgery. logMAR = logarithm of the minimum angle of resolution; Pre = preoperative; M = month(s); Y = year(s). The *p*-value was obtained from the paired *t*-test.

**Figure 2:**
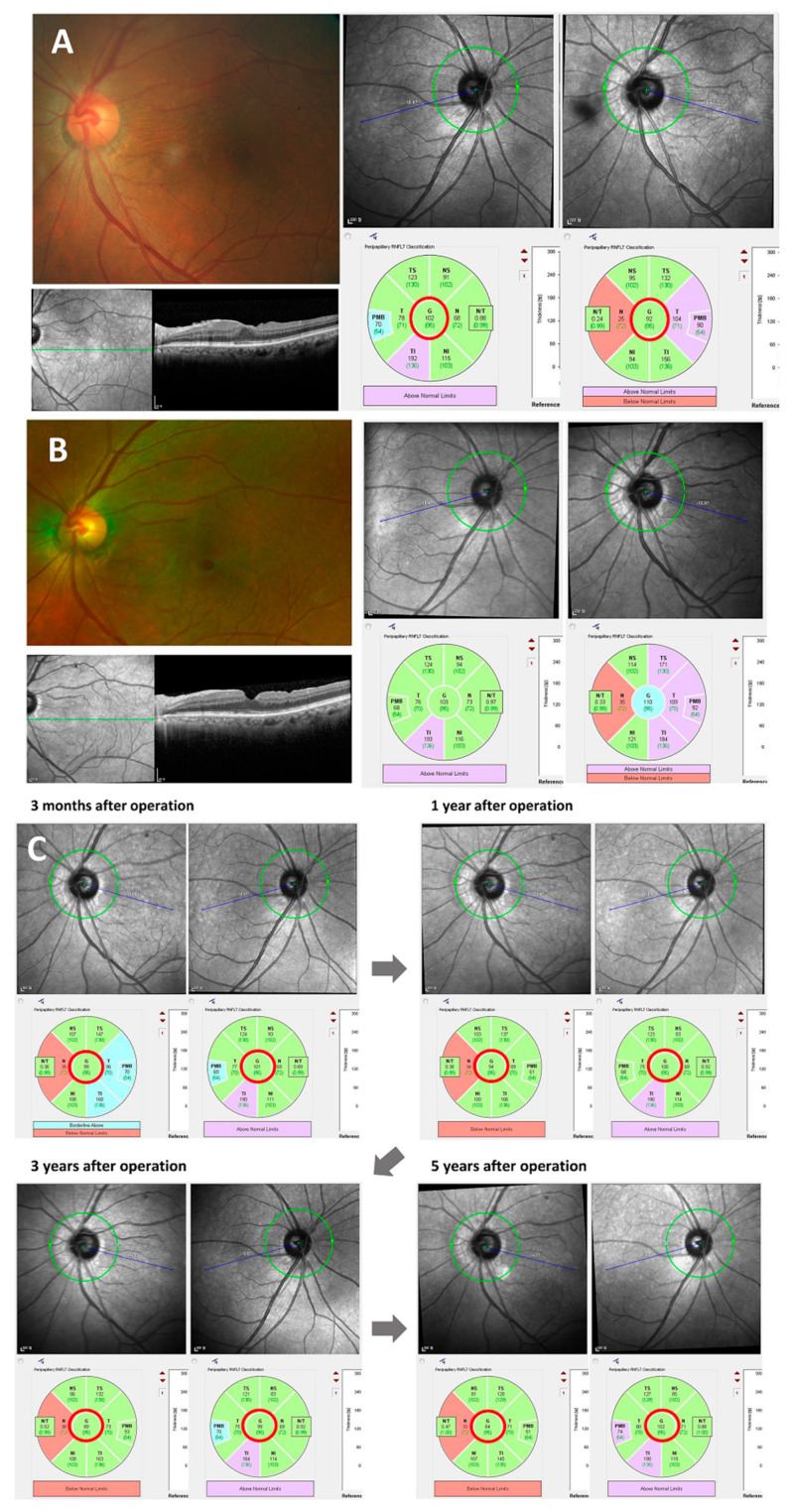
A representative case showing a gradual decrease in the peripapillary retinal nerve fiber layer thickness after vitrectomy for epiretinal membrane. A 70-year-old woman had an idiopathic epiretinal membrane (ERM) in her left eye, as shown in a preoperative fundus photo and optical coherence tomography (OCT) image (**A**). The baseline central macular thickness (CMT) and global peripapillary retinal nerve fiber layer thickness (GpRNFLT) were 362 and 92 μm, respectively (**A**). The patient underwent vitrectomy and ERM removal. After surgery, the ERM was peeled off, as presented by the fundus photo and OCT scan (**B**). The best corrected visual acuity improved from 0.7 to 0.9 one month after surgery. The CMT decreased to 344 μm, and the GpRNFLT increased to 110 μm. The GpRNFLT gradually decreased during follow-up, and it was 84 μm 5 years after surgery, as shown in serial OCT scans (**C**). However, the temporal change in GpRNFLT of the fellow eye was insignificant. The GpRNFLT of the fellow eye was 102 μm at baseline and the last visit.

Thirty eyes from 30 patients were included in this study and a total nineteen fellow eyes were identified as the control group. The demographic characteristics of the study population are presented in Table 1. Among them, 24 were female (80.0%), and the mean age at the time of the surgery was 65.4 ± 7.2 years (range, 46–76). The average baseline BCVA was 0.28 ± 0.24 logMAR (Snellen equivalent, 20/40), and the vision significantly improved to 0.12 ± 0.09 logMAR (Snellen equivalent, 20/25, *p* < 0.001) 1 year after surgery; the improvement continued to 0.06 ± 0.08 logMAR (Snellen equivalent, 20/23, *p* < 0.001) 5 years after surgery (Figure 1). None of the study eyes showed an IOP increase exceeding 21 mmHg during the follow-up, and no significant IOP changes compared to the baseline were identified during any visit after surgery (all *p*s > 0.05). In addition, there were no significant differences in IOP between the study eyes and their fellow eyes at baseline or at any time point after surgery (n = 19, all *p*s > 0.05). A representative case is presented in Figure 2.

### OCT Analysis

**Figure 3:**
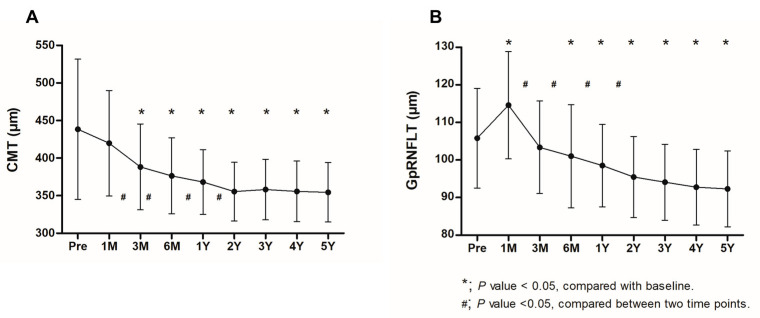
Temporal changes in the central macular thickness (CMT) and global peripapillary retinal nerve fiber layer thickness (GpRNFLT) after surgery (n = 30). (**A**) The patients with epiretinal membranes showed a gradual decrease in the CMT after vitrectomy and membrane peeling during follow-up. (**B**) The preoperative mean GpRNFLT of the study eyes was 105.8 ± 13.3 μm, and it significantly increased to 114.6 ± 14.3 μm 1 month after surgery (*p* < 0.001). After that, a gradual decrease continued, and a significant reduction was observed 6 months after surgery. An asterisk indicates a statistically significant difference from baseline. Data are presented as mean ± standard deviation. Pre = preoperative; M = month(s); Y = year(s). The *p*-value was obtained from the paired *t*-test.

The preoperative mean CMT of the study eyes was 438.4 ± 93.3 μm, which significantly decreased to 368.2 ± 43.1 μm (*p* < 0.001) 1 year after surgery and 354.6 ± 39.6 μm (*p* < 0.001) 5 years after surgery. The CMT of the operated eyes continuously decreased after surgery (Figure 3). Compared to before surgery, the CMT significantly reduced 3 months after surgery (*p* < 0.001), and this reduction continued until 5 years after surgery. At baseline, the mean CMT was 438.4 ± 93.3 μm and significantly decreased to 388.3 ± 57.1 μm, 368.2 ± 43.1 μm, and 354.6 ± 40.0 μm during the postoperative 3 months, 1 year, and 5 years (all *p*s < 0.001), respectively. There were significant differences in CMT between 1 and 3 months, 3 and 6 months, 6 and 12 months, and 1 and 2 years after surgery (all *p*s < 0.05). The baseline aMT of the study eyes was 354.4 ± 44.9 μm, which significantly decreased to 312.7 ± 15.9 μm (*p* < 0.001) 1 year after surgery and 304.7 ± 14.8 μm (*p* < 0.001) 5 years after surgery. The mGCCT at the time of the initial visit was 140.7 ± 29.5 μm and significantly decreased to 101.3 ± 7.7 μm (*p* < 0.001) 1 year after surgery and 100.7 ± 9.7 μm (*p* < 0.001) 5 years after surgery. The temporal changes of the aMT and mGCCT are presented in Table 2.

**Figure 4:**
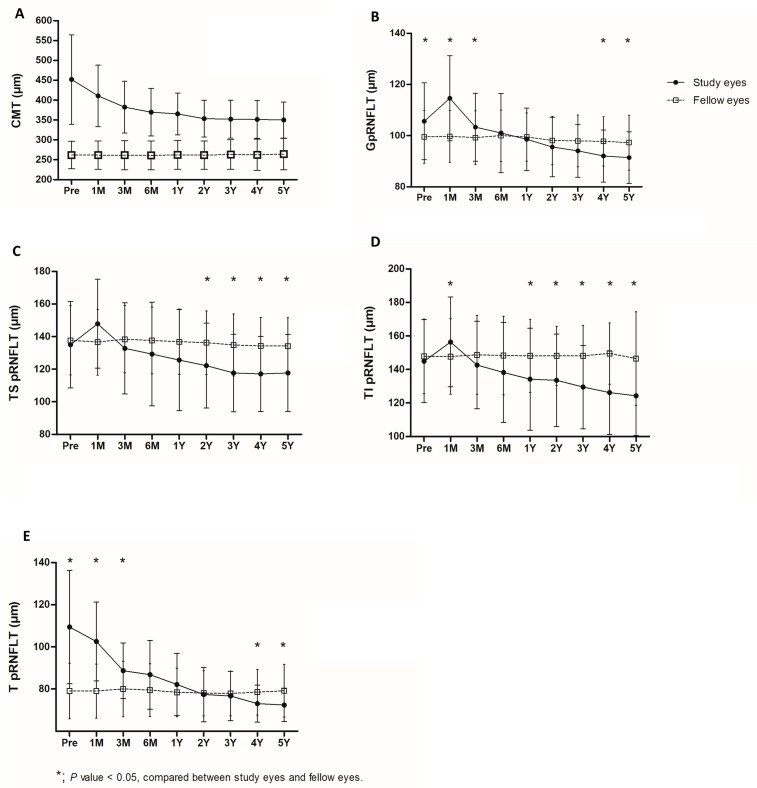
Comparison of the temporal changes in the central macular thickness (CMT) (**A**), global peripapillary retinal nerve fiber layer thickness (**B**), peripapillary retinal nerve fiber layer thickness in the temporal superior (**C**), temporal inferior (**D**), and temporal (**E**) sectors of the eyes that underwent vitrectomy for epiretinal membrane removal and their fellow eyes (n = 19). (**A**) The study eyes showed a gradual decrease in CMT after vitrectomy during the follow-up. The average CMT of the study eyes was significantly greater than that of their fellow eyes at all time points. The global pRNFLT and those of the TS and TI sectors in the study eyes showed a transient increase 1 month after surgery, followed by a continuous gradual decrease(**B**–**D**). Meanwhile, the temporal pRNFLT of the study eyes showed a continuous reduction immediately after surgery (**E**). During the follow-up, the temporal changes in pRNFLT in the fellow eyes were not significant. However, pRNFLT of the global and temporal areas (TS, TI, and T) continued to decrease. Therefore, significant differences in the pRNFLTs of these areas between the study and fellow eyes were confirmed. Data are presented as mean ± standard deviation. CMT, central macular thickness; pRNFLT, peripapillary retinal nerve fiber layer thickness; G, global; TS = temporal superior; TI = temporal inferior; T = temporal; Pre = preoperative; M = month(s); Y = year(s). *p*-values were obtained using the Wilcoxon signed-rank test.

The baseline global peripapillary RNFL thickness of the study eyes was 105.8 ± 13.3 μm, and it significantly increased to 114.6 ± 14.3 μm 1 month after surgery (*p* < 0.001 Compared to the baseline examination, the average global peripapillary RNFL thicknesses were significantly reduced to 101.0 ± 13.7 μm (*p* = 0.04), 98.5 ± 11.0 μm (*p* = 0.002), and 92.3 ± 10.1 μm (*p* < 0.001) during the postoperative 6 months, 1 year, and 5 years, respectively. There were significant differences in global peripapillary RNFL thicknesses between 1 and 3 months, 3 and 6 months, 6 and 12 months, and 1 and 2 years after surgery (all *p*s < 0.05). Temporal changes in the peripapillary RNFL thicknesses are shown in Table 3. The peripapillary RNFL thicknesses of the global and all six sectors, except the temporal sector, showed a transient increase after the surgery, and a continuous decrease was reported during the follow-up. Meanwhile, the temporal peripapillary RNFL thickness showed a continuous reduction immediately after surgery. The peripapillary RNFL thicknesses of the global and temporal areas (TS, T, and TI) continued to decrease significantly and showed a significant reduction compared to the baseline 2 years after surgery (all *p*s < 0.05). The peripapillary RNFL thicknesses of the nasal area (NI, N, NS) also decreased after a temporary increase immediate after the operation. However, it was not definite and did not show a significant change compared to baseline in the peripapillary RNFL thicknesses of the nasal area. A comparison of the CMTs and peripapillary RNFL thicknesses of the global and temporal areas of the study and fellow eyes was conducted, and the results are shown in Figure 4.

The CMTs of the study eyes were significantly greater than those of their fellow eyes at baseline and any point after surgery. The peripapillary RNFL thicknesses of the global and temporal areas of the operated eyes decreased continuously and were significantly thinner than those for the fellow eyes 4 and 5 years after surgery. However, there was no significant difference between the peripapillary RNFL thicknesses of the nasal areas of the study and fellow eyes 4 and 5 years after surgery.

The correlation analysis was performed using Pearson correlation. The BCVA did not show a significant correlation with the peripapillary RNFL thickness of any sector in all time points during the follow-up (all *p*s > 0.05). The CMT showed a significant correlation with peripapillary RNFL thickness in the temporal areas (T, TS, and TI) during the observation. Moreover, the aMT consistently showed a significant positive correlation with peripapillary RNFL thickness in the temporal sector between baseline and 3 years after operation: baseline (r = 0.582, *p* = 0.001), 1 month after surgery (r = 0.400, *p* = 0.029), 3 months after surgery (r = 0.380, *p* = 0.038), 6 months after surgery (r = 0.375, *p* = 0.041), 1 year after surgery (r = 0.402, *p* = 0.028), 2 years after surgery (r = 0.588, *p* = 0.001), and 3 years after surgery (r = 0.466, *p* = 0.009). The aMT and mGCCT have a very strong positive correlation at all time points (*p*s ranged 0.001–0.025). Furthermore, a constant and meaningful correlation was identified between mGCCT and peripapillary RNFL in the temporal sector during the observation: baseline (r = 0.572, *p* = 0.003), 1 month after surgery (r = 0.643, *p* = 0.002), 3 months after surgery (r = 0.527, *p* = 0.017), 6 months after surgery (r = 0.549, *p* = 0.012), 1 year after surgery (r = 0.508, *p* = 0.010), 2 years after surgery (r = 0.504, *p* = 0.010), 3 years after surgery (r = 0.489, *p* = 0.013).

## 4. Discussion

We investigated the long-term outcomes after ERM surgery, including the BCVA, CMT, aMT, mGCCT, and peripapillary RNFL thickness. Vision improved 3 months after surgery, and visual gain was maintained for more than 5 years, consistent with the results of previous studies [9,10]. The CMT also significantly decreased 3 months after surgery, and the reduction in macular thickness was sustained during follow-up, similar to the findings of previous reports [9,10]. Few studies have evaluated the temporal changes in peripapillary RNFL thickness after ERM surgery. In this study, the peripapillary RNFL thicknesses temporarily thickened 1 month after surgery, followed a continuous decrease. The global peripapillary RNFL thickness for the temporal areas continued to decrease after a transient increase after surgery and showed significant thinning 1 year after surgery, which was maintained until 5 years postoperatively. Meanwhile, the peripapillary RNFL thicknesses of the nasal area showed a temporary increase after surgery; however, there was no significant difference from that before the surgery during long-term follow-up.

The aMT consistently showed a strong positive correlation with the peripapillary RNFL thickness in the temporal sector between baseline and 3 years after ERM surgery, as did mGCCT and temporal peripapillary RNFL thickness. The retinal thicknesses around the macula, such as CMT, aMT, and mGCCT, steeply decreased until 2 years after surgery and tended to maintain thereafter as shown in Table 2 and Figure 3. However, the peripapillary RNFL thickness in the temporal area seemed to constantly decrease after operation. Therefore, the peripapillary RNFL thicknesses in the temporal areas of the study eyes were significantly thinner than those of fellow eyes in control group 4 and 5 years after surgery (Table 3 and Figure 4). The aMT and mGCCT did not show a significant correlation with peripapillary RNFL thickness in the temporal sector 4 years and 5 years after surgery. This result may be due to the difference in thickness change between the macula and peripapillary area. In other words, although the peripapillary RNFL thickness decreased continuously, the reduction in the retinal thickness around the macula attenuated during the follow-up. Further studies may be needed to confirm the difference in the retinal thickness change between the macula and peripapillary area.

Several studies reported the impact of ERM on the thickness measurement of the macular area and peripapillary RNFL using OCT [22,23,24,25]. An ERM usually involves the macula, centers on the perifovea, and often extends to the peripapillary area. The ERM causes tangential traction and structural changes including retinal wrinkles or distortion. Therefore, there are concerns about the reproducibility and reliability of the retinal thickness measurement in ERM patients. Nam et al. revealed that the repeatability of the measurement of peripapillary RNFL thickness performed by SD-OCT was fair, even in patients who had an ERM with peripapillary involvement [25]. Some studies showed that the temporal and global peripapillary RNFL thicknesses were significantly higher than those of normal controls [23,24]. In particular, the thickening in temporal and global peripapillary RNFL was much higher in patients who had an ERM that extended to the peripapillary area [23]. Increased peripapillary RNFL thicknesses are explained by the tangential tractional force of the ERM on the retina [23,24]. The results from the previous reports are consistent with our data. The baseline peripapillary RNFL thickness in the temporal sector and global area was significantly higher than that of normal contralateral eyes in our study population, as shown in Figure 4.

The transient thickening of the peripapillary RNFL is thought to be related to postoperative inflammation after vitrectomy, as suggested in the research by Lim et al. [26]. They reported a temporary increase in peripapillary RNFL thickness in patients with intraocular lens dislocation who underwent vitrectomy and scleral fixation without macular manipulation during the immediate postoperative period. Several studies have reported peripapillary RNFL thinning in the temporal area after ILM peeling [17,18,19,20,21,27,28]. The gradual thinning of the peripapillary RNFL in the temporal area may be related to several surgical factors, including retinal toxicity from vital dyes, phototoxicity from endoillumination, IOP fluctuation during the surgery, and mechanical damage during membrane peeling [18,21]. If the retinal toxicity of a vital dye or photic injury was the main cause of the peripapillary RNFL thinning, a generalized reduction in peripapillary RNFL thicknesses in all sectors, rather than only the temporal areas, would be observed. However, there was no significant change in peripapillary RNFL thicknesses in the nasal area during the long-term follow-up. The IOP fluctuation was negligible because the surgery used the Constellation vitrectomy system, which can maintain the IOP during surgery [29]. Additionally, no significant IOP increase was reported postoperatively.

Some studies have reported that ILM peeling causes the loss of the basement membrane of the Müller cells [30,31]. Damage to the Müller cells may result in axonal transport change and apoptosis and atrophy of the ganglion cells, leading to visual deterioration [32]. Moreover, the superficial retinal vessels are prone to damage during ILM peeling, and this may lead to temporary ischemic changes in the GCCs, including the RNFL and GCL [13,14]. Scupola et al. showed a significant correlation between the area of RNFL swelling, which corresponded to the sites of surgical grasping during ILM peeling, and postoperative peripapillary RNFL thinning [20]. They suggested that mechanical damage due to surgical grasping of the ILM may have caused the peripapillary RNFL reduction after ERM surgery.

This study has some limitations. The study was retrospective and included a small number of patients. We evaluated only peripapillary RNFL thickness changes and did not perform functional examinations such as visual field test or microperimetry. Instead, we analyzed the correlation between visual acuity and peripapillary RNFL thickness. However, we did not find significant results. Some previous studies have suggested a correlation between anatomical changes and functional tests [21,27]. However, to the best of our knowledge, we conducted the longest follow-up study of peripapillary RNFL thickness changes after ERM surgery.

In conclusion, peripapillary RNFL thickness gradually decreased in the global and temporal areas in patients after ERM removal and ILM peeling during long-term follow-up. These results may be related to mechanical injury caused by ILM peeling. Further studies are required to investigate the relationship between structural changes and functional outcomes in more patients with ERM after surgery.

## Figures and Tables

**Table 1:** Demographic characteristics of patients with epiretinal membrane.

	Study Eyes (n = 30)	Fellow Eyes (n = 19)	*p* Value
Age (years, range)	65.4 ± 7.2 (46–76)	63.3 ± 7.3 (46–76)	0.333 *
Male (%)	6/30 (20.0%)	4/19 (21.1%)	0.601 †
Refraction (D, SE, range)	0.40 ± 1.00 (−2.13–2.63)	0.10 ± 1.30 (−3.63–2.00)	0.338 *
BCVA (logMAR, range)	0.28 ± 0.24 (0.05–1.00)	0.05 ± 0.09 (0.00–0.30)	0.001 §
Intraocular pressure (mmHg, range)	16.4 ± 2.8 (11–21)	16.9 ± 3.2 (12–21)	0.594 *
CMT (μm, range)	438.4 ± 93.3 (300–718)	261.8 ± 34.8 (217–324)	<0.001 *
Global pRNFLT (μm, range)	105.8 ± 13.3 (79–135)	99.5 ± 10.3 (75–114)	0.086 *

Data are presented as total no. (%) or mean ± standard deviation unless otherwise indicated. D, diopter; SE, spherical equivalent; BCVA, best-corrected visual acuity; logMAR, logarithm of the minimum angle of resolution; CMT, central macular thickness; pRNFLT, peripapillary retinal nerve fiber layer thickness. * *p-*values were obtained using an independent test. † *p-*values were obtained using Fisher’s exact test. § *p-*values were obtained using the Mann–Whitney U test.

**Table 2:** Temporal changes in central macular thickness (n = 30), average macular thickness (n = 30), and mean ganglion cell complex thickness (n = 25).

	CMT	*p*	aMT	*p*	mGCCT	*p*
Baseline	438.4 ± 93.3	N/A	354.4 ± 44.9	N/A	140.7 ± 29.5	N/A
1 month	419.8 ± 70.0	0.156	344.5 ± 22.5 *	<0.001	115.7 ± 10.8 *	<0.001
3 months	388.3 ± 57.1 *	<0.001	325.2 ± 17.7 *	<0.001	105.9 ± 8.9 *	<0.001
6 months	376.4 ± 50.5 *	<0.001	316.7 ± 16.9 *	<0.001	103.6 ± 7.9 *	<0.001
1 year	368.2 ± 43.1 *	<0.001	312.7 ± 15.9 *	<0.001	101.3 ± 7.7 *	<0.001
2 years	355.5 ± 39.1 *	<0.001	308.6 ± 15.4 *	<0.001	100.3 ± 8.10 *	<0.001
3 years	358.3 ± 40.2 *	<0.001	307.1 ± 15.1 *	<0.001	99.4 ± 6.8 *	<0.001
4 years	355.8 ± 40.4 *	<0.001	305.4 ± 14.1 *	0.002	100.8 ± 9.3 *	<0.001
5 years	354.6 ± 39.6 *	<0.001	304.7 ± 14.8 *	0.002	100.7 ± 9.7 *	<0.001

Data are presented as mean ± standard deviation (μm). CMT, central macular thickness; aMT, average macular thickness; mGCCT, mean ganglion cell complex thickness; N/A, not applicable. * Denotes a significant difference (*p* < 0.05). *p*-values were obtained using the paired *t*-test (compared with the baseline value).

**Table 3:** Temporal changes in peripapillary retinal nerve fiber layer thickness after surgery (n = 30).

	Global	*p*	TS	*p*	T	*p*	TI	*p*	NI	*p*	N	*p*	NS	*p*
Baseline	105.8 ± 13.3	N/A	133.9 ± 24.0	N/A	106.9 ± 27.8	N/A	144.4 ± 23.1	N/A	104.4 ± 17.5	N/A	70.8 ± 15.0	N/A	107.5 ± 19.4	N/A
1 month	114.6 ± 14.3 *	<0.001	146.2 ± 25.4 *	<0.001	101.2 ± 16.6	0.108	156.7 ± 23.5 *	<0.001	119.7 ± 19.6 *	<0.001	84.8 ± 20.0 *	<0.001	122.1 ± 27.4 *	<0.001
3 months	103.4 ± 12.3	0.258	132.1 ± 26.8	0.518	86.6 ± 13.0 *	<0.001	143.0 ± 23.6	0.631	112.1 ± 17.7 *	0.005	77.6 ± 15.4 *	0.003	111.7 ± 23.5	0.231
6 months	101.0 ± 13.7 *	0.040	129.4 ± 28.9	0.239	84.5 ± 15.4 *	<0.001	139.4 ± 25.9	0.148	110.7 ± 19.9	0.070	76.1 ± 16.2 *	0.009	109.6 ± 27.8	0.616
1 year	98.5 ± 11.0 *	0.002	126.2 ± 28.0	0.054	80.1 ±13.3 *	<0.001	135.6 ± 25.9 *	0.013	108.2 ± 16.0	0.169	73.6 ± 14.6	0.162	107.5 ± 25.6	0.993
2 years	95.5 ± 10.8 *	<0.001	123.0 ± 25.1 *	0.004	76.3 ± 12.3 *	<0.001	134.8 ± 24.5 *	0.004	108.6 ± 17.5	0.209	72.8 ± 13.7	0.335	102.3 ± 25.8	0.227
3 years	94.1 ± 10.1 *	<0.001	118.4 ± 23.8 *	<0.001	74.8 ± 11.7 *	<0.001	130.4 ± 22.6 *	0.001	108.4 ± 18.7	0.261	72.5 ± 14.2	0.447	100.9 ± 23.7	0.109
4 years	92.8 ± 10.1 *	<0.001	117.5 ± 23.3 *	<0.001	72.5 ± 9.7 *	<0.001	128.3 ± 23.1 *	<0.001	107.9 ± 20.6	0.304	72.1 ± 14.0	0.564	99.75 ± 24.4	0.055
5 years	92.3 ± 10.1 *	<0.001	118.4 ± 23.0 *	<0.001	71.8 ± 8.5 *	<0.001	126.5 ± 21.6 *	<0.001	107.4 ± 20.0	0.359	71.7 ± 15.5	0.710	100.3 ± 23.6	0.073

Data are presented as mean ± standard deviation (μm). TS, temporal superior; T, temporal; TI, temporal inferior; NI, nasal inferior; N, nasal; NS, nasal superior; N/A, not applicable. * Denotes a significant difference (*p* < 0.05). *p*-values were obtained using the paired *t*-test (compared with the baseline value).

## Data Availability

The data are not available for public access because of patient privacy concerns but are available from the corresponding author upon reasonable request.
